# Goal-Oriented Optimization of Dynamic Simulations to Find a Balance between Performance Enhancement and Injury Prevention during Volleyball Spiking

**DOI:** 10.3390/life11070598

**Published:** 2021-06-22

**Authors:** Dhruv Gupta, Cyril J. Donnelly, Jody L. Jensen, Jeffrey A. Reinbolt

**Affiliations:** 1 Mechanical, Aerospace and Biomedical Engineering, The University of Tennessee, Knoxville, TN 37996, USA; 2 Rehabilitation Research Institute of Singapore, Nanyang Technological University, Singapore 308232, Singapore; cyril.donnelly@ntu.edu.sg; 3 School of Human Sciences (Health and Sport Sciences), The University of Western Australia, Crawley, WA 6009, Australia; 4 Department of Kinesiology and Health Education, The University of Texas at Austin, Austin, TX 78712, USA; jljensen@austin.utexas.edu

**Keywords:** volleyball, hitting performance, shoulder torques, performance–injury balance, optimization, participant-specific modeling, dynamic simulations

## Abstract

Performance enhancement and injury prevention are often perceived as opposite sides of a coin, where focusing on improvements of one leads to detriment of the other. In this study, we used physics-based simulations with novel optimization methods to find participant-specific, whole-body mechanics of volleyball spiking that enhances performance (the peak height of the hitting hand and its forward velocity) while minimizing injury risk. For the volleyball spiking motion, the shoulder is the most common injury site because of the high mechanical loads that are most pronounced during the follow-through phase of the movement. We analyzed 104 and 209 spiking trials across 13 participants for the power and follow-through phases, respectively. During the power phase, simulations increased (*p* < 0.025) the peak height of the hitting wrist by 1% and increased (*p* < 0.025) the forward wrist velocity by 25%, without increasing peak shoulder joint torques, by increasing the lower-limb forward swing (i.e., hip flexion, knee extension). During the follow-through phase, simulations decreased (*p* < 0.025) peak shoulder joint torques by 75% elicited by synergistic rotation of the trunk along the pathway of the hitting arm. Our results show that performance enhancement and injury prevention are not mutually exclusive and may both be improved simultaneously, potentially leading to better-performing and injury-free athletes.

## 1. Introduction

Enhancing arm swing performance without increasing injury risk during a volleyball spike are two pillars supporting the long-term success of any volleyball attacker. The hitting hand height and forward velocity are important determinants of performance during the power phase of this motion, while the most common site of injury is the shoulder joint during the follow-through phase (i.e., eccentric loading) [1,2,3]. Maximizing performance and minimizing injury risk is the goal of coaches and athletes alike, but it is difficult to achieve when using a heuristic or intuitive experimental approach. Achieving a balance between performance and injury risk is considered as a difficult training task, as increased performance is coupled with kinematics that are known to underpin musculoskeletal injuries. Kinematic changes are non-linearly associated with both performance [4,5,6] and shoulder injury risk [6,7,8,9,10] during the volleyball spiking motion. For this reason, optimal kinematics may potentially increase performance and decrease injury risk simultaneously [6]. The downstream effects of identifying optimal kinematics could significantly advance the field of athletic training and rehabilitation, and potentially lead to longer injury free careers at a high level of performance for the athletes.

High magnitude and high frequency loads at the shoulder during the spiking motion are known risk factors related to overhead hitting shoulder injuries [3,11,12,13,14]. Because of the joint complexity, a shoulder and surrounding soft tissue injuries can manifest in many ways. These injuries include, but are not limited to, rotator cuff muscle/tendon injury [1,2,15], suprascapular neuropathy [16,17], and impingement injuries [18]. The major cause of injury during volleyball spiking is believed to be high eccentric stress on the relatively smaller rotator cuff muscles which are used as ‘brakes’ to decelerate the fast-moving arm during the follow-through phase [1,2]. Reducing eccentric mechanical load on the shoulder may reduce injury during volleyball spiking.

Modified kinematics to enhance performance and reduce injury risk have been proposed with limited scope [6]. Seminati et al. (2015) compared traditional spiking style to a completely different hitting style known as the backswing style, which resulted in a potentially safer path of humeral rotation while increasing ball velocity. However, researchers did not measure or predict the shoulder forces and torques during spiking that would result in fewer injuries. Additionally, established athletes cannot be expected to learn a totally new and different hitting style, mooting the practical applicability of this study. Other kinematic variables like hip angular velocity [5] and orientation of trunk and pelvis [4] have been related to performance. Kinematics like humeral and scapular range of motion [7,9] and humeral rotation velocity [6] have been associated with shoulder injury. These studies help us understand various performance and injury aspects of volleyball spiking, but they are limited in scope since they fail to characterize the entire kinematic and kinetic chain of the hitting motion underlying the injury mechanisms.

Optimal movement of body segments far from the injury site can reduce loads at the primary injury site [19]. Musculoskeletal modeling combined with simulation allows for “what if” questions to be asked that are difficult to answer experimentally [20,21]. Optimization methods have been used to find optimal whole-body kinematics during high velocity sporting tasks to reduce the risk of injury [19,22,23,24], but never to enhance sporting performance. This shortfall is because typical simulation generating methods depend on tracking given kinematics, and performance optimization requires knowledge of unknown kinematics.

In this study, we employed a novel technique of tracking a hypothetical target trajectory that depicts a greater increase in the performance parameters. This tracking forces simulations to increase performance and create changes in all body segments. The simulations are subject to anatomic and dynamic constraints; thus, the resulting kinematics remain feasible. Another challenge for optimization problems is balancing between performance and injury prevention goals. To address this challenge, we used a novel approach of adding goal-oriented step functions in the cost function of the optimization problem. These terms generate simulations that can achieve both a desired performance and an injury prevention goal. During each optimization, we ensured the optimal motion was not too different from the original motion, increasing the likelihood that the optimal motions can be learned, increasing the practical applicability of our results. In this study, we use computational musculoskeletal modeling and simulation methods to find participant-specific optimal whole-body kinematics for volleyball spiking that simultaneously enhances performance and reduces shoulder injury risk. We hypothesized that there will be an increase in performance parameters (peak height and forward velocity of hitting wrist), without an increase in the injury risk parameters (peak shoulder joint torques) pre-to-post optimization in the power phase. In addition, we hypothesized that there will be a decrease in injury risk parameters (peak shoulder joint torques) pre-to-post optimization in the follow-through phase.

## 2. Materials and Methods

### 2.1. Experimental Data

We used data from a pool of data collected for a previous study [25]. Ethical approval for the study was obtained from the Institutional Review Board of The University of Texas at Austin (Study 2014-09-0072). Participants gave written informed consent before participating in the study. The data was derived from the spiking motion of all right-handed players from a dataset 15 skilled volleyball hitters (7 men, 6 women, age: 23.3 ± 3.22 years, height: 1.9 ± 0.08 m, mass: 77.5 ± 10.45 kg, minimum level of experience: National Collegiate Volleyball Federation Division 1, minimum level of training: 12 h per week). Data were collected in a laboratory using a 10 MX camera motion capture system (Vicon Motion Systems, Oxford, UK). The athletes hit the ball under the constraints of three different instructions: (1) use of the participant’s natural hitting style, (2) use of maximal knee flexion in mid-flight, and (3) use of no knee flexion in mid-flight. The laboratory was set up to resemble a standard volleyball court, with the net set at the standard height, a marked center line, and a marked 3 m attack line. The ball was placed at each participant’s preferred hitting height [25]. In total, 209 trials were used for simulation purposes in the power phase and 249 trials in the follow-through phase.

### 2.2. Musculoskeletal Model

We used a force/torque actuated upper and lower body musculoskeletal model (simtk.org). This model combines a generic lower limb and trunk model [20,26] with an upper extremity model [27]. For computational efficiency, we locked the subtalar and metatarsophalangeal (mtp) joints and removed the spline function in knee flexion angle. Additionally, we locked the wrist joints because there was only one marker on the hand. The model had 29 degrees of freedom, 6 for pelvis, 5 for each leg (3 for hip, 1 for knee and 1 for ankle), 3 for the trunk and 5 for each arm (3 for shoulder, elbow flexion and pronation supination). This generic model was scaled to each participant in OpenSim 3.3 [20]. An inverse kinematics analysis generated the generalized coordinates for each spiking trial. The three shoulder coordinates were the plane-of-shoulder-elevation angle, shoulder elevation angle and shoulder rotation angle, consistent with the ISB recommendations [28].

### 2.3. Two-Step Optimization Process

**Figure 1:**
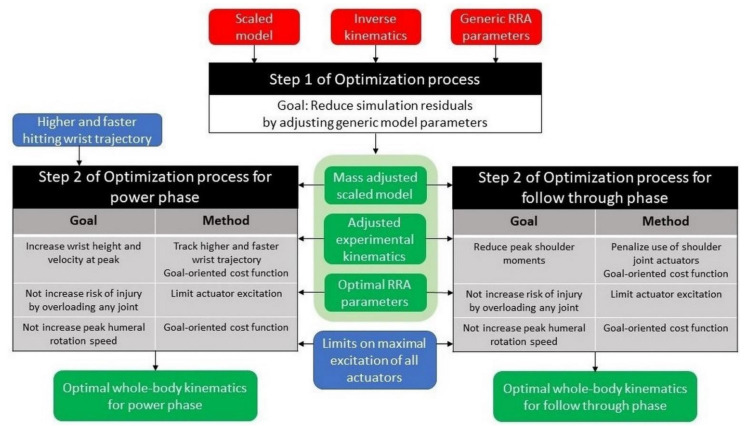
The two-step optimization process to find optimal whole-body kinematics.

Inverse kinematics was followed by a 2-step optimization process, similar to the process used by Donnelly et al. (2012), to find optimal whole-body kinematics. Each step of this process is an outer level optimization [29]. Outer level optimization is a routine that optimizes the parameters of the simulation generated by the inner level optimization process. For this study, the residual reduction algorithm (RRA) in OpenSim was used. Hence, each cost function evaluation of the outer level optimization is a new simulation. Figure 1 depicts the two-step optimization process. Simulations were not time normalized before executing the RRA as RRA performs computations each millisecond and this was held constant for all athletes.

#### 2.3.1. Step 1 of the Optimization Process

The first outer level optimization ensures that the experimental data are accurately represented in the simulations by optimizing the human-defined parameters of the model to reduce simulation residuals [19,22,29]. These parameters are the maximal force/torque capacity of each of the 29 actuators ($R^{max}$ and $T^{max}$) and the 29 weights at which the joint coordinates are tracked in the cost function of RRA ($w_{\ddot{q}}$).

Step 1 RRA cost function:

(1) $$\underset{x^{R},\;x^{T}}{\min}\left[\overset{n_{q}}{\underset{i=1}{\sum }}w_{\ddot{q_{i}}}{\left({\ddot{q}}_{i}^{exp}-{\ddot{q}}_{i}^{sim}\right)}^{2}+\overset{6}{\underset{j=1}{\sum }}{\left(\frac{R_{j}\left(x_{j}^{R}\right)}{R_{j}^{max}}\right)}^{2}+\overset{n_{T}}{\underset{k=1}{\sum }}{\left(\frac{T_{k}\left(x_{k}^{T}\right)}{T_{k}^{max}}\right)}^{2}\right]$$

where, $x^{R}$ and$\;x^{T}$ are the excitation of the force and torque actuators. ${\ddot{q}}^{exp}\;$and ${\ddot{q}}^{sim}$ are the experimental and simulation generated accelerations and $w_{\ddot{q}}$ are the tracking weights of the $n_{q}=29$ degrees of freedom. $R\left(x^{R}\right)$ refers to the 6 residuals (three forces and three torques). $T\left(x^{T}\right)$ represents the torques generated by the $n_{T}=n_{q}-6$ torque actuators. $R^{max}$ and $T^{max}$ are maximum capacities of the residuals and joint torque actuators, respectively. The outer level optimization optimizes parameters of the RRA optimization to ensure that the simulation kinematics $(q^{sim}$) are dynamically consistent with the experimental kinematics $(q^{exp}$) and the residuals (R) are near zero.

Step 1 outer-level optimization cost function:

(2) $$\underset{w_{\ddot{q}},R^{max},\;T^{max}}{\min}\overset{n_{f}}{\underset{i=1}{\sum }}\left[\begin{array}{c} W_{pelvis}\sum_{j=1}^{6}{\left(q_{ij}^{exp}-q_{ij}^{sim}\right)}^{2}+W_{non-pelvis}\sum_{j=7}^{n_{q}}{\left(q_{ij}^{exp}-q_{ij}^{sim}\right)}^{2}\\ +W_{R}\sum_{k=1}^{6}R_{ik}{}^{2} \end{array}\right]$$

where, $W_{pelvis}=500$, $W_{non-pelvis}=1000$ and $W_{R}=1000$ were manually chosen weights of terms in the cost functions and $n_{f}$ are the number of frames over which the optimization was performed. The optimization was performed from the start of forward motion of the elbow to hit the ball and continued until 30 ms after the peak shoulder torques in the follow-through phase, where eccentric forces are at their highest [1,2,3]. RRA uses 1 ms increments when generating a simulation, and 30 ms was sufficient to capture peak shoulder torques during the follow-through phase of the simulation. This process provided a mass-adjusted participant-specific scaled model, participant-specific RRA parameters, and adjusted experimental kinematics. The kinematics were adjusted to be dynamically consistent and reduce pelvis residuals, which represent the errors and assumptions made in the modeling process [19,29]. Once we obtained optimal model parameters for each participant using the first outer level optimization, these calculated parameters were kept constant for all trials of the same participant. We accepted the simulations only if the absolute value of average residual forces was <1 N, absolute value of average residual moments <5 Nm, kinematic translation error <3 cm, and kinematic rotational error <5°. These error parameters are consistent with the parameters used in previous studies [19,22,24]. The outer level optimization is required to handle a large number of design variables, thus, we used gradient-based optimization for this process.

#### 2.3.2. Step 2 of the Optimization Process

Once optimal, participant-specific model parameters were obtained in Step 1 of the optimization process, they were used again for the second step. For Step 2 of the optimization process (or the second outer level optimization), the goal was to find simulations that satisfy the performance and injury-related goals. The power phase of overhead hitting involves both performance enhancement and injury prevention aspects, while the follow-through phase is concentrated upon injury prevention. The goal of the optimization changes between the power phase and the follow-through phase, thus Step 2 of the optimization was performed separately for each phase.

##### Step 2 of the Optimization Process for the Power Phase

Power phase starts when the elbow begins forward motion following maximum cocking and lasts until the last frame before ball contact [30]. The goal of the optimization process in the power phase is to find optimal whole-body kinematics that enhance performance without increasing the risk of injury. This entails satisfying two performance goals and three injury prevention goals simultaneously. The performance goals were: (1) increase the peak height of the hitting wrist, and (2) increase the forward velocity of the hitting wrist when it is at its peak. The injury prevention goals were: (1) prevent peak shoulder torques from increasing, (2) prevent overload of any other joint torque in the whole-body kinetic chain, and (3) do not allow peak humeral angular velocity magnitude (HAVM) to increase. Increasing the performance parameters during the power phase can increase humeral angular velocity, with the potential to cause injury in the follow-through phase. Additionally, increasing performance parameters while mitigating shoulder loads during the power phase can increase loads on other joints of the body, putting them at a risk of injury. For these reasons, we did not allow increases in peak humeral angular velocity and restricted the allowable increase in joint torques across all joints. To inhibit overload, a limit on the maximal allowable excitations was applied on each joint coordinate’s actuator. During the power phase, this limit represented the peak torque of a joint coordinate actuator in a trial plus one standard deviation of peak torque for the specified coordinate across all trials (specific to gender and hitting condition. For the three coordinate actuators of the shoulder joint, the limit was the peak torque in each trial as we did not want to increase the peak shoulder torques. To ensure that the peak HAVM did not increase, but the performance parameters did increase, we use a goal-oriented cost function for the outer level optimization.

Humeral angular velocity magnitude (HAVM) is the vector sum of the velocities of the three shoulder coordinates. To ensure that the optimization does not increase the peak HAVM, we added a goal-oriented term in the cost function of the outer level optimization to account for it.

(3) $$HAVM\;term=\{\begin{array}{cc} peak\;HAVM, & \;\;peak\;HAVM>peak\;HAVM_{exp}\\ 0, & \;\;otherwise \end{array}$$

This goal-oriented cost functions meant that for the simulations generated during the optimization process, if the peak HAVM was greater than the experimental peak HAVM, the HAVM term took the value of the peak HAVM. Otherwise, it took a value of 0. This step function in the outer level optimizer cost function encourages the optimization to favor the simulations that do not allow an increase in the peak HAVM.

Similarly, goal-oriented cost function terms were added to promote an increase in peak height of the hitting wrist and the forward velocity of the hitting wrist at the moment of peak height. We calculated the percent increase in the peak height of wrist (pIH), and the percent increase in the forward velocity of the wrist at its peak height (pIV), and designed a wrist cost function term.

(4) $$Wrist\;term=\{\begin{array}{cc} \left(pIH\times pIV\right)+10, & \;\;pIH<0\;and\;pIV<0\\ -\left(pIH\times pIV\right)-10, & \;\;pIH>0\;and\;pIV>0\\ pIH\times pIV, & \;\;otherwise \end{array}$$

Using a product of pIH and pIV in the cost function ensures that the outer level optimization does not favor either increase in peak height or velocity over the other. The notation of ‘±10′ in the wrist term encourages the optimizer to favor simulations which increase both peak height and velocity at peak height of the wrist, simultaneously. At the same time, this wrist term ensures that the optimization does not favor the simulations that decrease either or both performance parameters. The final cost function of the outer level optimization, that is, the Step 2 outer-level optimization cost function for power phase, was a weighted sum of the HAVM term and the wrist term.

(5) $$Cost\;function=W_{HAVM}\times HAVM\;term+W_{w}\times Wrist\;term$$

where, $W_{HAVM}=0.005$ and $W_{w}=0.1$ were manually chosen. The terms of the cost function have step functions in them, thus gradient-based optimization cannot be used for the outer level optimization. We used a simulated annealing algorithm [31] because it can handle non gradient-based cost functions and it is a global, not local, optimization algorithm.

In addition to the terms in the outer level cost function, the simulation generating process (RRA) controlled by the outer level needs to be made capable of finding new motions that meet the performance and injury prevention goals. To allow RRA to find a new optimized motion, tracking weights of all upper limb coordinates, lower limb coordinates and trunk rotation about the vertical axis were multiplied by a factor between 0 and 1. This factor was one of the design variables of the outer level optimization that controls the inner level RRA. Tracking of the pelvic coordinates was not reduced since pelvic movement directly affects residuals. Additionally, trunk and pelvis vertical excursion affects the hang-time of the volleyball athletes [25,32]. Hang-time is a period of time when the athlete is around the peak of his/her flight and the head and trunk of the athlete stays at a near constant vertical height [33]. Studies have shown that athletes tend to swing later when they use hang-time, increasing their decision-making time before ball contact, which has the potential to enhance performance [25,34]. For this reason, we chose not to change the hang-time during the simulation process by not reducing the tracking of the coordinates that influence hang-time.

To promote RRA to find motions that increase the peak height of the hitting wrist and its forward velocity, we added a tracking task in the cost function of RRA. This tracking task penalized the difference between the trajectory of the hitting wrist and a hypothetical target trajectory that was p% higher and v% faster than the experimental trajectory of the hitting wrist. The values of p and v were design variables of the outer level optimization. It is possible that values of p and v are so high that the hypothetical trajectory may not be anatomically or dynamically feasible, but tracking it pushes the trajectory of the hitting wrist in the simulation towards higher performance goals. This can force a change in all segments of the body but since the simulation is still subject to anatomic and dynamic constraint, the resulting kinematics will still be feasible. The power phase begins when the elbow starts to move forward, and it is unreasonable to increase the height and forward velocity of the hitting wrist at the start of power phase. Hence, the target trajectory was designed such that the increase in height was maximal at the peak (p%) and 0 at the lowest point of the experimental trajectory of the hitting wrist. At each time frame i, increase in height of the target trajectory compared to the experimental trajectory was $\left(x_{pi}\times p\right)\%$, where,

(6) $$x_{pi}=\frac{height\;at\;frame\;i-\min height}{\max height-\min height}$$

The same implementation was used for the forward velocity increase as well. At each time frame i, the increase in forward velocity of the target trajectory compared to the experimental trajectory was $\left(x_{vi}\times v\right)\%$, where,

(7) $$x_{vi}=\frac{forward\;velocity\;at\;frame\;i-\min forward\;velocity}{\max forward\;velocity-\min forward\;velocity}$$

**Figure 2:**
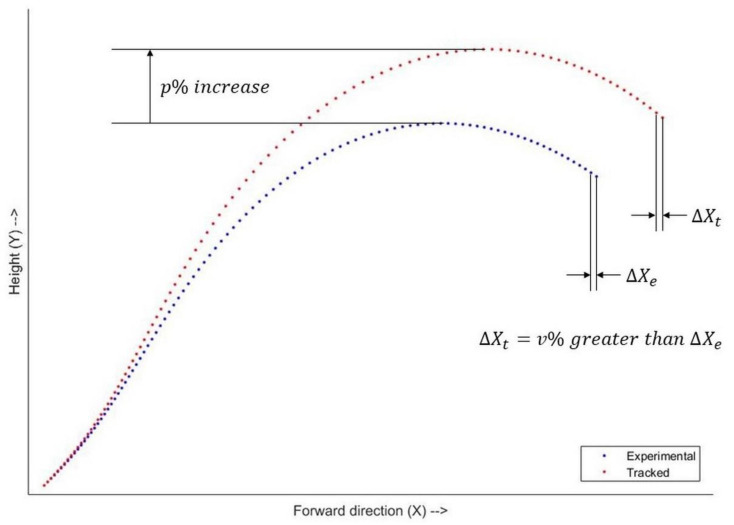
Sagittal plane view of experimental and target trajectory of the wrist joint center. Each marker represents a time frame. Both trajectories have the same lowest point and lowest forward velocity. The peak of the target trajectory is p% higher than that of the experimental trajectory. In the experimental trajectory, the highest forward velocity typically occurred just before ball contact, or the last frame of the power phase. At the last frame, the forward velocity (difference between forward position of consecutive frames) of the target trajectory is v% greater than that of the experimental trajectory.

Figure 2 shows an example of the experimental and the target trajectory.

Step 2 RRA cost function for power phase:

(8) $$\underset{x^{R},\;x^{T}}{\min}\left[\begin{array}{c} \sum_{i=1}^{8}w_{\ddot{q_{i}}}{\left({\ddot{q}}_{i}^{exp}-{\ddot{q}}_{i}^{sim}\right)}^{2}+\sum_{i=9}^{n_{q}}f_{\ddot{q}}w_{\ddot{q_{i}}}{\left({\ddot{q}}_{i}^{exp}-{\ddot{q}}_{i}^{sim}\right)}^{2}+\sum_{j=1}^{6}{\left(\frac{R_{j}\left(x_{j}^{R}\right)}{R_{j}^{max}}\right)}^{2}\\ +\sum_{k=1}^{n_{T}}{\left(\frac{T_{k}\left(x_{k}^{T}\right)}{T_{k}^{max}}\right)}^{2}+w_{p}{\left(Y_{WJC}^{tgt}-Y_{WJC}^{sim}\right)}^{2}+w_{v}{\left(X_{WJC}^{tgt}-X_{WJC}^{sim}\right)}^{2} \end{array}\right]$$

where, k=1 to 8 are for the 6 pelvis and 2 trunk coordinates whose tracking weights were not reduced. Tracking weights of all other coordinates were multiplied by a factor $f_{\ddot{q}}$. The weights $w_{p}=1000$ and $w_{v}=1000$ was manually chosen. The last two terms of the cost function represent the difference between the X and Y positions of the simulation hitting wrist joint center ($X_{WJC}^{sim}$ and $Y_{WJC}^{sim}$) and those of the target trajectory of the hitting wrist joint center ($X_{WJC}^{tgt}$ and $Y_{WJC}^{tgt}$). This trajectory was p% higher and v% faster than the experimental trajectory of the hitting wrist. $f_{\ddot{q}},\;p$ and v were the design variables of the outer level optimization. Again, optimal values of $w_{\ddot{q}},\;R^{max}$ and $T^{max}$ were determined in Step 1 of the optimization process.

Even though the goal-oriented cost function terms encourage the optimization process to generate simulations of specific biomechanical properties, it is possible that the optimization process fails. For example, it is possible that a simulation achieves an extremely high percentage increase in the forward velocity at peak height (say pIV=20) by reducing the peak height (e.g., pIH=−1). This scenario will lead to a value of −20 for the Wrist term (Equation (4)). The optimizer will prefer this to a 1 percent increase in both peak height and forward velocity, which leads to a value of −11 for the Wrist term (Equation (4)). It is also possible that even though residual reduction is a part of the cost function, the simulation still generates high residuals if it reduces the cost from other terms. To address this issue, we accepted a post optimization simulation only if it met all the following acceptance criteria (a) increased peak height of the hitting wrist, (b) increased forward velocity at peak height of the hitting wrist, (c) decreased peak HAVM, and (d) the absolute values of average residual forces and residual moments are lower than 5 N and 5 Nm, respectively.

##### Step 2 of the Optimization Process for the Follow-through Phase

The follow-through phase lasts from the first frame after ball contact until 30 ms post peak shoulder torques, where eccentric forces are at their highest [1,2,3]. The goal of this optimization is to find optimal whole-body kinematics that reduce peak external rotation joint torques and the risk of shoulder injury. This entails satisfying three injury prevention goals simultaneously: (1) reduce peak shoulder torques, (2) prevent overloading of any other joint of the body and (3) prevent peak HAVM from increasing. Reducing loads on the shoulder during the follow-through phase, when rapid deceleration of the arm is required, can add loads on other joints along the kinematic and kinetic chain. For these reasons, we did not allow increases in peak HAVM and restricted the allowable increase in joint torques across all joints similar to power phase. To reduce the peak torques of the shoulder joint coordinates in the follow-through phase, we designed a new goal-oriented cost function term, the shoulder torque (ST) term, one for each direction (positive and negative) of each of the three shoulder coordinates.

For $i^{th}$ shoulder coordinate, the ST term in $j^{th}$ direction,

(9) $$ST\;term_{ij}=f\left(x\right)=\{\begin{array}{cc} rM_{ij}+1, & \;\;rM_{ij}>0.9\\ rM_{ij}, & \;\;otherwise \end{array}$$

where, $rM_{ij}$ is the ratio of the peak shoulder torque in the $j^{th}$ direction of the $i^{th}$ shoulder coordinate to the experimental peak shoulder torque of that coordinate (i=1,2,3 and j=1,2). This implementation encourages the optimization to favor simulations that allow for at least 10% reduction in both peak positive and peak negative torques across each of the three shoulder coordinates. The final Step 2 outer-level optimization cost function for the follow-through phase was a weighted sum of the HAVM term and the ST terms.

(10) $$Cost\;function=W_{HAVM}\times HAVM\;term+W_{ST}\sum_{i=1}^{3}\sum_{j=1}^{2}ST\;term_{ij}$$

where, $W_{HAVM}=0.01$ and $W_{ST}=1$ were manually chosen. Like the power phase, the terms of the cost function of outer level optimization in the follow-through phase have step functions in them. Hence, we again used a simulated annealing algorithm for the outer level optimization.

Similar to the power phase, we reduced the tracking weights of all coordinates, except the pelvic coordinates by a factor between 0 to 1. This factor was a design variable of the outer level optimization. To ensure that the RRA reduced the peak shoulder torques, the penalty of using the torque actuators of the three shoulder coordinates was decreased by multiplying the maximum capacity of the shoulder coordinates actuators by a factor between 0 and 1. Each coordinate was assigned a factor and these factors were design variables of the outer level optimization.

Step 2 RRA cost function for follow-through phase:

(11) $$\underset{x^{R},\;x^{T}}{\min}\left[\begin{array}{c} \sum_{i=1}^{6}w_{\ddot{q_{i}}}{\left({\ddot{q}}_{i}^{exp}-{\ddot{q}}_{i}^{sim}\right)}^{2}+\sum_{i=7}^{n_{q}}f_{\ddot{q}}w_{\ddot{q_{i}}}{\left({\ddot{q}}_{i}^{exp}-{\ddot{q}}_{i}^{sim}\right)}^{2}+\sum_{j=1}^{6}{\left(\frac{R_{j}\left(x_{j}^{R}\right)}{R_{j}^{max}}\right)}^{2}\\ +\sum_{k=1}^{3}{\left(\frac{T_{k}\left(x_{k}^{T}\right)}{f_{k}T_{k}^{max}}\right)}^{2}+\sum_{k=4}^{n_{T}}{\left(\frac{T_{k}\left(x_{k}^{T}\right)}{T_{k}^{max}}\right)}^{2} \end{array}\right]$$

where, k=1,2,3 are the three shoulder coordinates. $f_{\ddot{q}},f_{1},f_{2}$ and $f_{3}$ are design variables of the outer level optimization. Optimal values of $w_{\ddot{q}},\;R^{max}$ and $T^{max}$ were determined in the Step 1 of the optimization process.

Similar to the power phase, we accepted a post-optimization simulation only if it met all of the following acceptance criteria; (a) all six ST terms were less than 0.9, implying that both peak positive and peak negative torques across all three shoulder coordinates were reduced by at least 10%, (b) decreased peak HAVM, and (c) the absolute value of average residual forces and residual moments were lower than 5 N and 5 Nm, respectively.

### 2.4. Post-Hoc Analyses

For both the power and follow-through phases of volleyball spiking, we wished to find the joint coordinates that elicited changes in the hitting arm motion, ultimately affecting the changes in performance or shoulder injury risk parameters—these were termed critical joint coordinates [19]. Critical joint coordinates are those, other than the hitting arm, that changed more than two standard deviations above the mean change of all coordinates for each trial. Since each participant had multiple trials, we also computed the “consistent critical coordinates”. These were operationally defined as the joint coordinates that were critical for at least 40% of the trials of each individual participant. This helped us find participant-specific optimal kinematic changes. To understand the general pattern of kinematic changes that elicited the change in performance and injury prevention parameters, we also computed the coordinates that were consistently critical for at least 40% of the participants. The threshold of 40% was chosen as it helped us balance between too few coordinates to explain the mechanism and too many that would indicate trial specific changes rather than a general pattern. This threshold is consistent with methods used in previous literature [22,24]. All computations were performed using MATLAB (R2017a, The MathWorks Inc., Natick, MA, USA, 2000) at the Texas Advanced Computing Center (TACC) at The University of Texas at Austin.

To statistically test our hypotheses, we compared changes in performance and injury prevention parameters in power and follow-through phases, and we compared the peak HAVM, and the peak torques of the shoulder coordinates pre-to-post optimization. All torque values were normalized to body mass and body height. For the power phase, we also compared the peak height of the hitting wrist and the forward velocity at its peak height pre-to-post optimization. We used Hierarchical Linear Modeling (HLM) as the statistics tool, with participants as level 2 units and pre-vs-post optimization as level 1 variables (α = 0.025, since our hypotheses are directional). HLM removes the variance accounted by the level 2 units and focuses on differences in the variables of interest caused solely by level 1 variables, the optimization process. In other words, variance in the data due to participant-specific differences like height, weight, hitting position, age, experience, etc. are treated as covariates and their influence removed thus allowing for the variance of the optimization procedure to be analyzed [25,35]. All statistical tests were performed in R 3.3.1 (R Core Team, 2016, R: A language and environment for statistical computing. R Foundation for Statistical Computing, Vienna, Austria. URL https://www.R-project.org/, accessed on 21 May 2020).

## 3. Results

**Figure 3:**
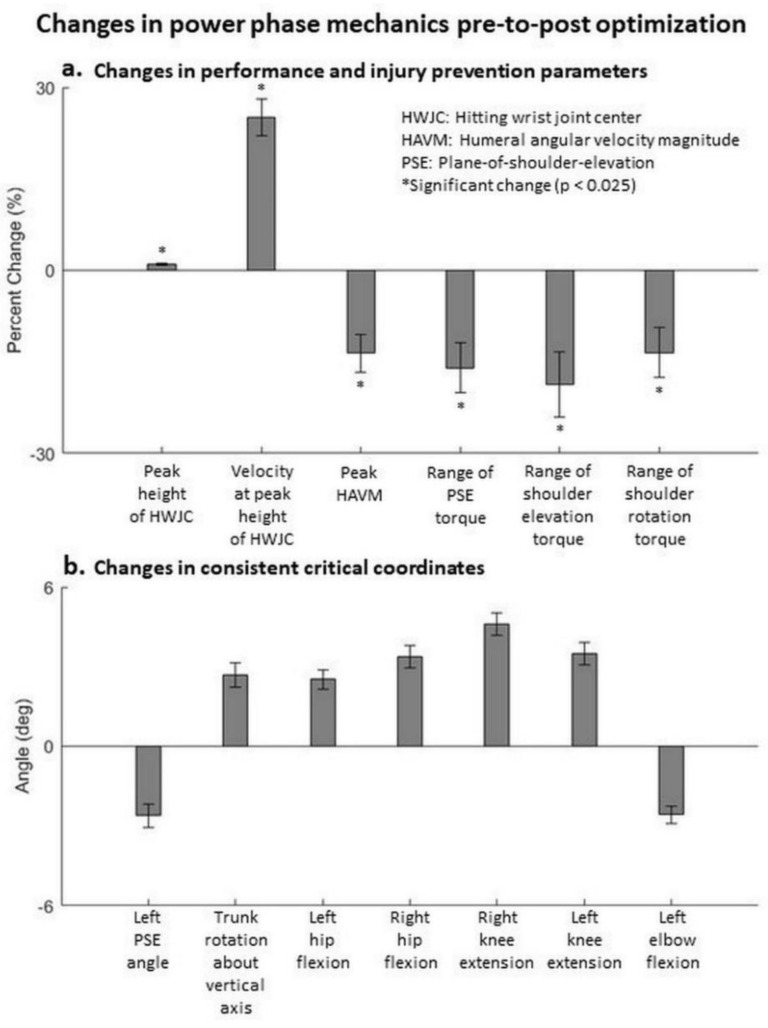
Changes in the power phase pre-to-post optimization. (**a**) Change in performance and injury prevention parameters. Mean ± standard error values across all participants are depicted. Note that in the statistical analyses, hierarchical linear modeling effectively compares trials of each participant pre-to-post optimization independent of other participants, rather than comparing all trials of all participants pre-to-post optimization. (**b**) Coordinates that were consistently critical for at least 40% of the participants. These coordinates represent a general pattern that elicit the changes in performance and injury prevention parameters.

For the power phase (104 accepted trials), the mean peak height of the hitting wrist significantly increased (*p* < 0.025) by 1%. The mean forward velocity of the hitting wrist at its peak height significantly increased (*p* < 0.025) by 25%. Peak HAVM decreased (*p* < 0.025) pre-to-post optimization (Figure 3a). At the same time point, none of the peak torques at the shoulder increased pre-to-post optimization (Figure 3a). These results support our first hypothesis. Seven critical joint coordinates for at least 40% of the participants contributed to the observed changes in performance (Figure 3b). The general kinematic pattern used to enhance performance without increasing the risk of shoulder injury can be described as the forward swinging of the legs with increased hip flexion and knee extension. Video S1 shows the pre- and post-optimization kinematics of a trial that follows the general pattern. Supplementary Materials Figure S1 shows that hip flexion angles of each leg were consistently critical for 91.7% (right) and 41.7% (left) of the participants, while knee extension angles of each leg were consistently critical for 41.7% (right) and 66.7% (left) of the participants. At least one of these four coordinates was consistently critical for all the participants, and at least three were consistently critical for 58.3% of the participants.

**Figure 4:**
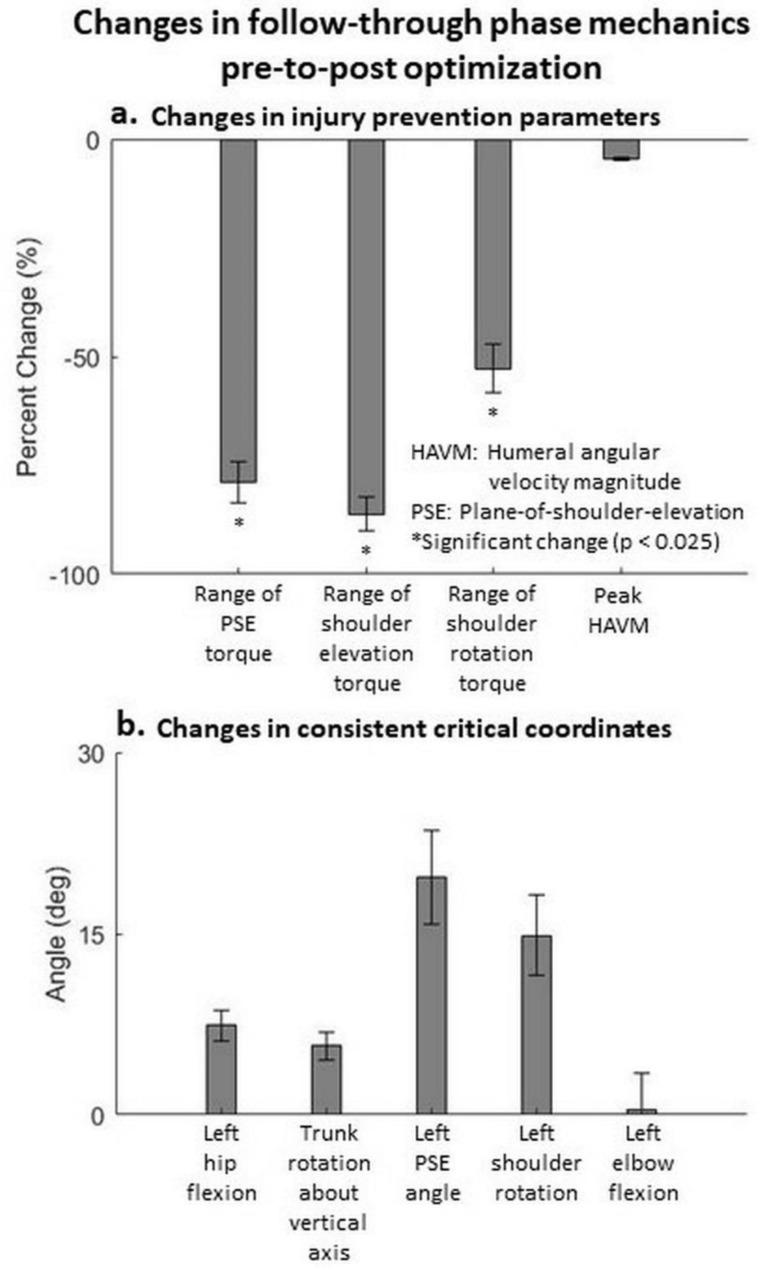
Changes in follow-through phase pre-to-post optimization. (**a**) Change in injury prevention parameters. Mean ± standard error values across all participants are depicted. Note that in the statistical analyses, hierarchical linear modeling effectively compares trials of each participant pre-to-post optimization independent of other participants, rather than comparing all trials of all participants pre-to-post optimization. (**b**) Coordinates that were consistently critical for at least 40% of the participants. These coordinates represent a general pattern that elicit the changes in the injury prevention parameters.

For the follow-through phase (209 accepted trials), mean peak joint torques at the shoulder coordinates decreased significantly (*p* < 0.025). The mean peak HAVM reduced pre-to-post optimization, however, this decrease was not significant (Figure 4a). The mean decreases in the range of torques across plane-of-shoulder-elevation angle, shoulder elevation angle and shoulder rotation angle coordinates were 80%, 88% and 56% (average 75%), respectively. These results support our second hypothesis. Five critical coordinates across at least 40% of the participants were observed (Figure 4b). These results show that the trunk rotation with the hitting arm is the primary kinematic pattern that reduces the peak shoulder torques. This is accompanied by increased left hip flexion and increased left plane-of-shoulder-elevation angle (bringing the left arm forward) pre-to-post optimization. Video S2 shows the pre- and post-optimization kinematics of a trial that follows the general pattern. Figure S2 shows that at least one out of these three coordinates was consistently critical for all the participants, at least two in 84.6% of the participants, and trunk rotation about vertical axis was consistently critical in 69.2% of the participants. It is also interesting to note that for certain trials, this mechanism of trunk rotating with the hitting arm manifested itself in a different way. Video S3 shows the experimental and the optimal kinematics for the follow-through phase of a trial from participant 8. For this trial, the critical coordinates were all in the sagittal plane (trunk forward rotation, right hip flexion and right ankle dorsiflexion). Closer observation of this trial revealed that the follow-through motion of the arm in the experimental motion was primarily in the sagittal plane. Consequently, the trunk rotated in the same direction to unload the shoulder and the right hip flexion aided conservation of angular momentum.

## 4. Discussion

Efforts to enhance performance do not have to be at the expense of injury. The results of our goal-oriented optimization of dynamic simulations demonstrate the potential of performance improvements with the likelihood of reduction of injury risk. For the volleyball spike, our simulations found optimal participant-specific, whole-body kinematic patterns that improved performance, without increasing injury risk at the shoulder or any other joint along the kinetic chain. These findings support our hypotheses that the performance parameters can increase without increasing the injury risk parameters pre-to-post optimization in the power phase, and injury risk parameters can decrease pre-to-post optimization in the follow-through phase. We found the key critical joint coordinates (called consistent critical coordinates), at both participant (Figures S1 and S2) and group level (Figure 3 and Figure 4), eliciting the changes in performance and injury prevention parameters for power phase and the follow-through phase of the hitting motions.

Our novel approach of tracking hypothetical performance trajectories successfully enabled the optimization to find emergent kinematics with better performance parameters. Our goal-oriented terms in the cost function enable the optimization to find a balance between performance enhancement and injury prevention goals. Additionally, this goal-oriented optimization gives athletes and coaches the flexibility to focus on specific performance and injury prevention goals. For example, if an athlete needs to mainly reduce injury risk, the performance parameters can be changed, removed, or activated only if the performance drops, similar to the HAVM term that only activates when the peak HAVM is higher than its experimental peak. Additionally, the cost function term addressing the injury prevention term could be made more important by additional weight or by forcing a decrease in peak shoulder torques greater than 10% (the value used in this study). Similarly, changes can be made to favor increases in performance parameters, based on the needs of the athletes and coaches.

Our study had some limitations. First, some goal-oriented terms in the outer level cost functions force the optimization to terminate once the goal is met, rather than at the global minimum. For example, the HAVM term only ensured that the peak HAVM did not increase but it did not encourage the outer level optimization to decrease the peak HAVM pre-to-post optimization. We acknowledge that this is a limitation, but it means that it is possible to have a movement pattern that is even better than the ones determined in this study. Nonetheless, our optimizations led to significantly enhanced performance and reduced injury risk parameters. Goal-oriented optimization helps achieve the performance-injury prevention balance and allow for athlete-specific biomechanical needs, an advantage that outweighs its limitations. Second, we performed the outer level optimization on the power and follow-through phases separately. This separation may introduce discontinuities between the optimal kinematics at the end of power phase and the start of the follow-through phase. It was necessary for us to perform the two optimizations separately as the goals of each optimization were different. Additionally, separate optimizations also enable coaches to focus of specific phases of the movement, ones that they think are more important for a given athlete. Further research is needed to integrate goals of different phases of movement into a single outer level optimization, while keeping the computational costs reasonable. Third, we used ideal force/torque actuators, rather than muscles, to drive our models. This choice does not affect the ability to find the optimal kinematics and the underlying mechanics. A future step would be to include muscle forces and identify specific muscles that drive the changes in performance enhancement and injury risk reduction to further assist the design of training and rehabilitation protocols. Fourth, the total number of accepted optimal simulations was different for power and follow-through phases. This difference resulted from more simulations violating the acceptance criteria of the power phase than the follow-through phase. More research needs to be done to make all cost function terms work together for higher numbers of accepted simulations. Even though we rejected some simulations, we were still able to generate a large number (313) of optimal whole-body, participant specific simulations. Despite limitations, we were able to generate participant-specific, whole-body simulations that achieve a balance between performance enhancement and injury prevention and understand the underlying mechanism.

During the power phase, we found forward swing of the lower body is the generalized mechanism driving the increase in the performance parameters without increasing the risk of shoulder injury. Since the athletes are in the air without externally applied torques, angular momentum is conserved and a hip forward swing needs to be balanced by a forward swing of the upper body, driving the hitting arm forward. The increased trunk vertical rotation pre-to-post optimization (consistently critical in 75% of the participants) aids the forward swing of the hitting hand (right), increasing the forward velocity. The increased knee extension during the power phase also drives the legs lower. The trajectory of whole-body center of mass cannot be changed mid-flight, thus the lowering of the legs drives the hitting hand higher. Our results are consistent with those observed in a previous study [32]. Additionally, since the lower-body forward swing, and not the shoulder, drives the hitting arm changes, the shoulder torques do not increase ensuring no added risk of shoulder injury. In this study, the percentage increase in peak height of the hitting wrist and the percent increase in its forward velocity at peak height were weighted equally in the cost function (wrist term). We still recorded that the percent increase in forward velocity (25%) was much higher than the percent increase in peak height (1%). This difference was likely because anatomical constraints on the arm length allowing for limited increases in the peak height. On the other hand, the forward velocity can be driven by forward swing of multiple lower body segments, so it has more freedom to increase. Noteworthily, maximum limits on joint torques were used to avoid overload, but the joints worked together synergistically to increase the forward velocity of the hitting wrist.

During the follow-through phase, we found trunk rotation in the direction of the hitting arm is the primary mechanism of reducing the risk of shoulder injury. Follow-through entails stopping the fast-moving arm. Rotation of the trunk with the arm transfers some of the load required to stop the arm from the shoulder to the larger trunk segment. The larger trunk muscles are better equipped to handle the load and save the smaller shoulder muscles that contract eccentrically to stop the fast-moving arm. Similar to the power phase, angular momentum is conserved in the follow-through phase, and it is achieved by an increased left hip flexion and increased left plane-of-shoulder-elevation angle pre-to-post optimization. These leg and arm angle increases are much higher than the vertical rotation of the trunk because the trunk is more massive and hence a greater displacement of the balancing segments is required to counter its angular momentum. This mechanism of trunk rotating with the hitting arm manifested itself in accordance with the experimental data. In a few trials, trunk forward flexion was a critical coordinate because the arm swing was primarily in the sagittal plane. The trunk rotation about the vertical axis was a consistent critical coordinate, while trunk forward flexion was only critical in a few trials because the majority of trials in this study featured the hitting arm crossing the front of the body, while only a few trials featured arm follow-through primarily in the sagittal plane. Importantly, the primary mechanism to unload the shoulder is same in both cases (trunk rotation in the direction of the arm follow-through). From an application point of view, athletes and coaches should focus on the consistent critical coordinates during training and rehabilitation in efforts to learn optimal movement patterns. Some of the techniques that could be employed to teach the optimal patterns are video based implicit learning [36,37], muscular training [38,39,40], and real-time feedback-based learning [41,42], and large scale interventions studies built on sound mechanics and behavior change principles [43]. The downstream effects could be potentially longer careers at a high level of performance for volleyball athletes.

## 5. Conclusions

This study made contributions towards both computational methods to find optimal movement patterns and identifying ways to achieve a balance between performance and injury risk for volleyball spikes. It lays the path to new training and rehabilitation protocols that could potentially transform long-term success of volleyball athletes. Our study may also lead to new avenues for finding this performance–injury balance in other sports.

## Data Availability

No new data were collected in this study. Data used in this study came from a pool of data collected for a previous study [25]. Data sharing is not applicable to this article.

## Supplementary Materials

The following are available online at https://www.mdpi.com/article/10.3390/life11070598/s1, Figure S1: Consistent critical coordinates for all the participants during power phase, Figure S2: Consistent critical coordinates for all the participants during follow-through phase, Video S1: Representation of the primary mechanism to enhance performance without increasing the risk of shoulder injury during power phase; Video S2: Representation of the primary mechanism to enhance performance without increasing the risk of shoulder injury during follow-through phase, Video S3: Experimental and optimal kinematics for the follow-through phase of a trial where the arm swing was primarily in the sagittal plane.
