# Supplementary figures and images for: Goal-Oriented Optimization of Dynamic Simulations to Find a Balance between Performance Enhancement and Injury Prevention during Volleyball Spiking
Source: Life (Basel). 2021 Jun 22;11(7):598. doi: 10.3390/life11070598 (PMC8303922; doi:10.3390/life11070598)

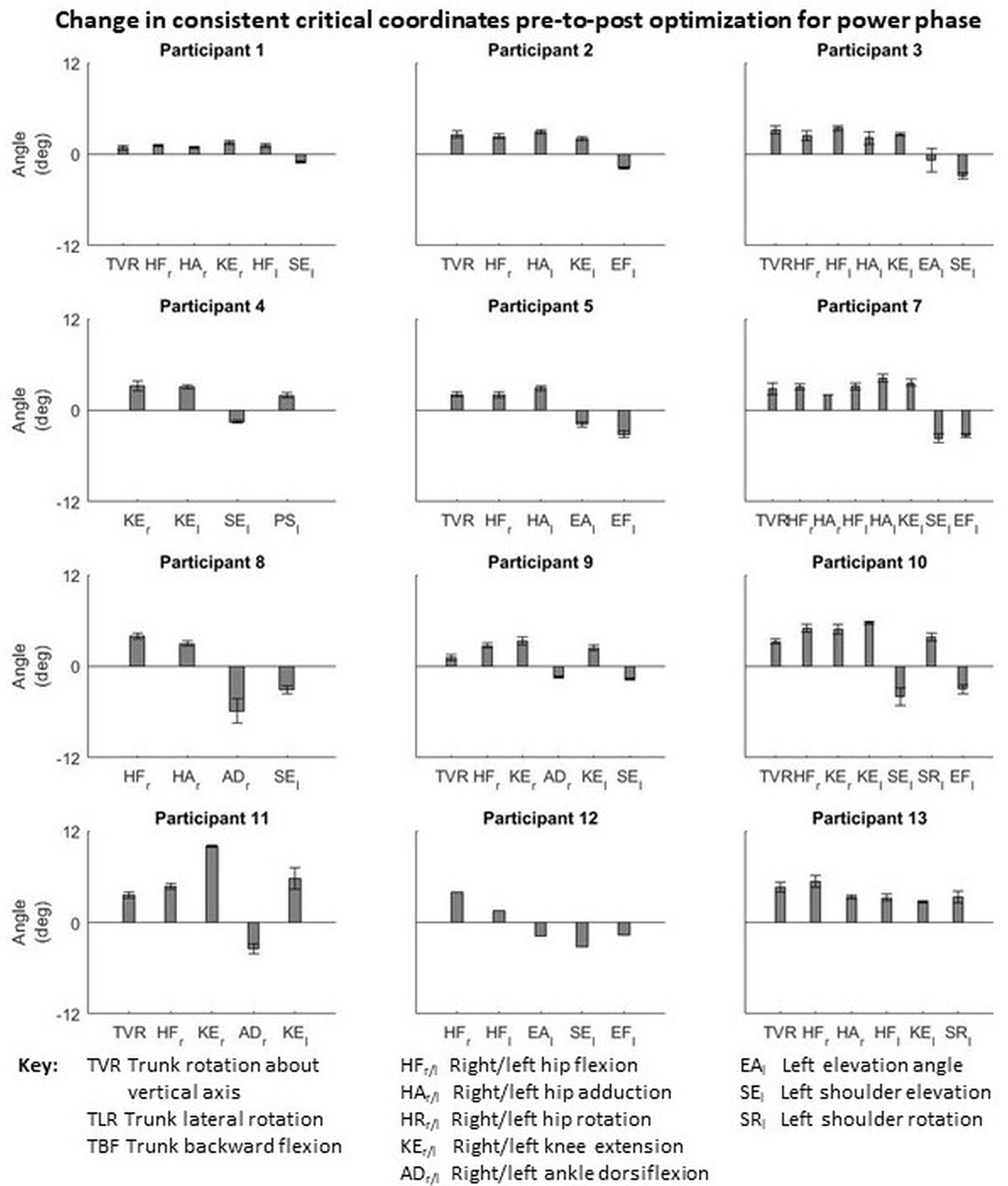

Supplement: Supplementary file 1 [file life-11-00598-s001.zip › Supplementary Data/Figure S1.jpg]

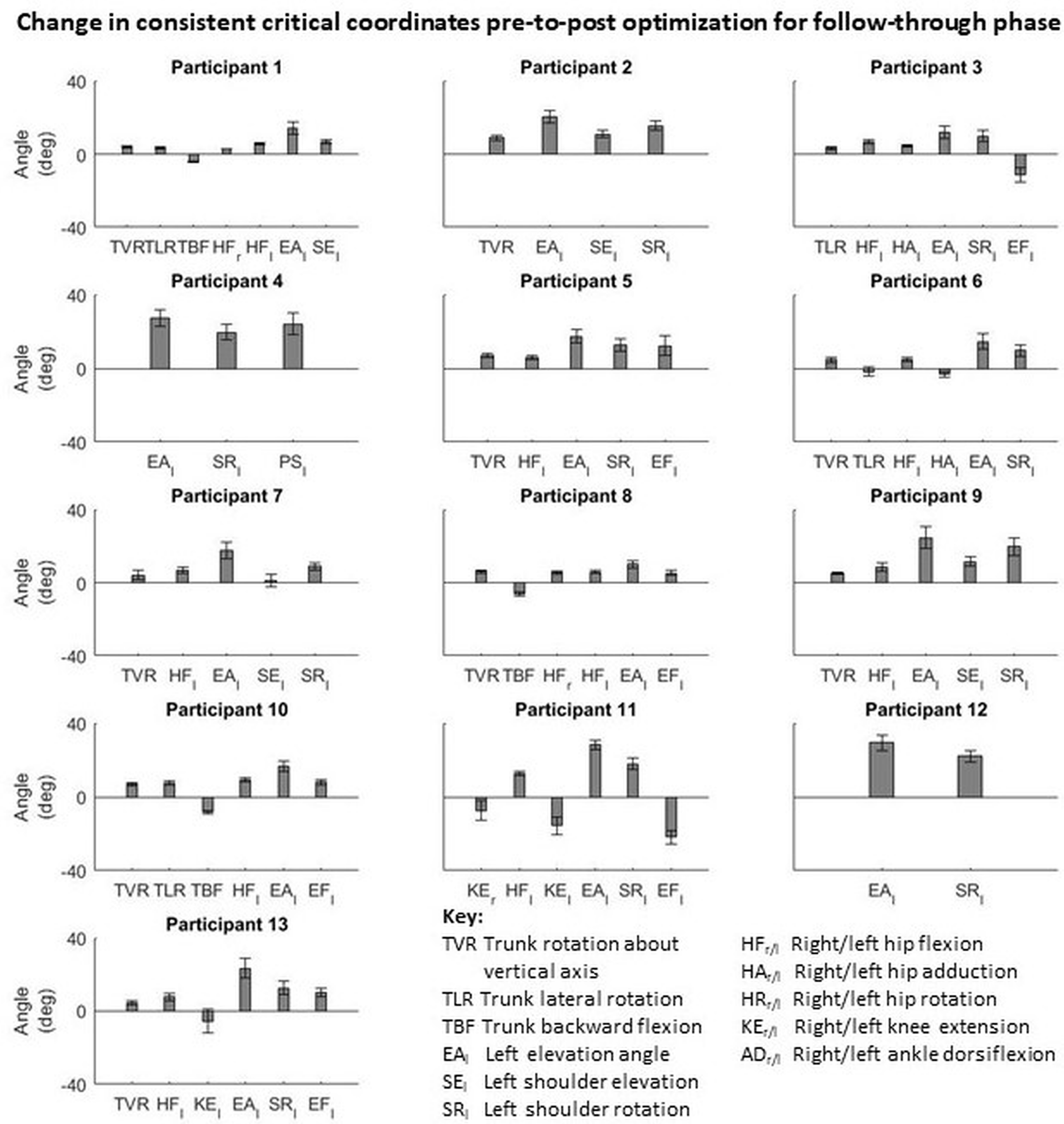

Supplement: Supplementary file 1 [file life-11-00598-s001.zip › Supplementary Data/Figure S2.jpg]
